# Supplementary material for: RNA-Binding Protein MAC5A Is Required for Gibberellin-Regulated Stamen Development
Source: Int J Mol Sci. 2022 Feb 11;23(4):2009. doi: 10.3390/ijms23042009 (PMC8874600; doi:10.3390/ijms23042009)
Supplement: Supplementary file 1 [file ijms-23-02009-s001.zip › ijms-1590411-supplementary.pdf]

International Journal of
Molecular Sciences

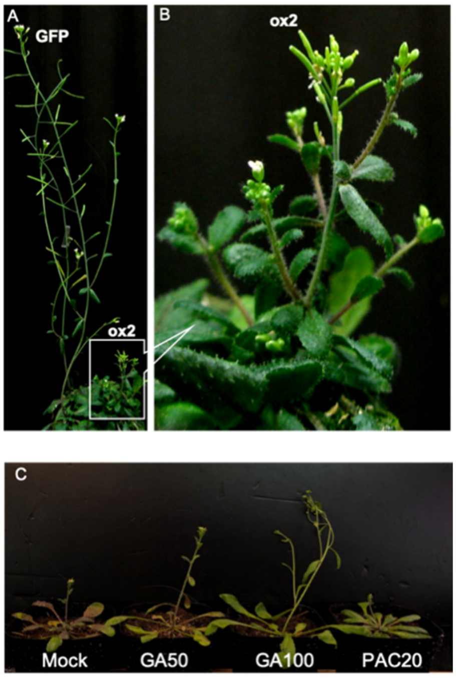

Supplemental Figure S1. Dwarf phenotype of plants overexpressing *MAC5A* can be partially alleviated by GA application. (**A**,**B**) Dwarf phenotype of ox2. GFP, plant overexpressing *GFP* alone. (**B**) The image of ox2 in panel A is enlarged in panel B. (**C**) Phenotype of ox2 after treatment with GA and PAC. GA50: 50 μM GA_3; GA100: 100 μM GA_3; PAC20: 20 μM PAC; Mock: control-treated without GA or PAC.

Supplemental Table S1. Primers used for plasmid construction and qRT-PCR.

Primer name	Primer sequence
MAC5A Fw *Hind*III	5'-AGGGAAGCTTTCTCTTATCCTCTGATTCG-3'
MAC5A Rv *Sma*I	5'-TACGCCCGGGCTTGTATTATACTAGGTTTACC-3'
MAC5A Fw *Kpn*I	5'-GAGTCGGTACCATGGCTCACAGAATAC-3'
MAC5A Rv *Spe*I	5'-GAGTCACTAGTCTACTGAGACGAACCAG-3'
MAC5A Fw *BamH*I	5'-AGGGGGATCC TCTCTTATCCTCTGATTCG-3'
GFP Fw *Xba*I	5'-AATCTAGAATGGTGAGCAAGGGCGAGGAGC-3'
GFP Rv *Sac*I	5'-AAGAGCTCTTACTTGTACAG CTCGTCCATG-3'
RBE Fw *Kpn*I	5'- GAGTCGGTACCATGATGGATAGAGGAGAATGCTTG -3'
RBE Rv *Spe*I	5'- GAGTCACTAGTGTTAACCTTAGGCGGATCAGCTCC-3'
RBE Fw *EcoR*I	5'- GAGTCGAATTCATGATGGATAGAGGAGAATGCTTG -3'
RBE Rv *BamH*I	5'-AGGGGGATCCGTTAACCTTAGGCGGATCAGCTCC-3'
AP1 qRT-PCR Fw	5'-TGCACCTGAGTCCGACGTCA-3'
AP1 qRT-PCR Rv	5'-TCCCCAAGATAATGCCTCTG-3'
AP2 QRT-PCR FW	5'-CTGCCGTAGTGGAGCCGGCA-3'
AP2 QRT-PCR RV	5'-TTCCCACAGTCCCAAATATG-3'
AP3 QRT-PCR FW	5'-TCAAGAAAGCACATGAGCTC-3'
AP3 QRT-PCR RV	5'-CGATCTCCTTCGTTGTGGTG-3'
PI qRT-PCR Fw	5'-TGCTAAGCATGAGAACCTTA-3'
PI qRT-PCR Rv	5'-TTCAAATGCCTGAGCTCCAG-3'
AG qRT-PCR Fw	5'-ATGCACAGTATTATCAACAAG-3'
AG qRT-PCR Rv	5'-CCATCAATTGCCTGTTGGAG-3'
KAO2 qRT-PCR FW	5'-ATCACCAGGTATGGGCGTAC-3'
KAO2 qRT-PCR RV	5'-GATACCAACAAAGGACTTCC-3'
GA3 qRT-PCR FW	5'-CTCTGTTCCAGTGGTACCAG-3'
GA3 qRT-PCR RV	5'-ACCATGGCCTCTTTGGCAGT-3'
GA2OX6 qRT-PCR FW	5'-GAACATCGGGTTCAACGGCG-3'
GA2OX6 qRT-PCR RV	5'-TTCGTCGCCGAGCTGAACTTG-3'
GA2OX2 qRT-PCR FW	5'-ACCTGAACCGTACGGTTATG-3'
GID1 C qRT-PCR FW	5'-ATTGAGAGCAAGACAGTGGTTC-3'
GID1 C qRT-PCR RV	5'-CTAAGCAAATTAGTTTGGCG-3'
RGA qRT-PCR FW	5'-AAGGTCGATTGTCCAACCACGG-3'
RGA qRT-PCR RV	5'-GTCCATGTTACCTCCACCGTC-3'
RGL2 qRT-PCR RV	5'-AGAGGATACGGAGAAACATG-3'
RGL2 qRT-PCR FW	5'-GAACGAGAAGCTGGTAGTGG-3'

Note: DNA sequences underlined are the sites recognized by enzymes.
